# Physical activity and depressive symptoms among Chinese adults a cross sectional analysis

**DOI:** 10.3389/fpsyg.2026.1989343

**Published:** 2026-10-27

**Authors:** Meng Xu, Kebing Yuan, Xupeng Zhang

**Affiliations:** 1 Department of Physical Education, Nanjing University of Aeronautics and Astronautics, Nanjing, China; 2 Faculty of Health Sciences and Sports, Macao Polytechnic University, Macao, China

**Keywords:** CES-D, China, cross-sectional study, depressive symptoms, physical activity, survey data

## Abstract

**Objective:**

To examine the association between total physical activity and depressive symptoms among Chinese adults, including possible departures from a linear dose–response relationship.

**Methods:**

We analyzed the 2024 Chinese National Sports and Society Survey, which obtained 11,385 adult responses across 31 provincial-level regions of mainland China. The complete-case sample comprised 10,663 adults. Activity duration was confirmed against the questionnaire as a weekly total and combined using intensity-specific MET coefficients. The primary outcome was the five-category ten-item depressive-symptom score (10–50). We fitted sampling-weighted estimating equations with an observation-level sandwich variance, categorical models and a two-part restricted cubic spline model. A study-specific recoded screening outcome was exploratory. Models adjusted for nonlinear age, sex, education, household registration, marital status, income and chronic disease.

**Results:**

Relative to inactivity, adjusted symptom-score differences were −3.11 (95% CI −5.94 to −0.27) for low, −3.41 (−6.13 to −0.69) for moderate and −2.20 (−4.96 to 0.57) for high activity. Low and moderate activity did not differ clearly (moderate minus low: −0.30, 95% CI −1.38 to 0.78). The primary nonlinear-term Wald test gave P = 0.034, but evidence weakened with three knots (*P* = 0.309) and exclusion above the 95th activity percentile (*P* = 0.063). All primary exploratory screening prevalence-ratio intervals included 1.

**Conclusion:**

Low and moderate activity were associated with lower continuous depressive-symptom scores than inactivity. The possible nonlinear pattern depended on modeling choices and did not establish an optimal dose or a stable J-shaped relationship. High activity was not shown to be more harmful than inactivity. Activity volume is distinct from intensity, and inactivity is not a measure of prolonged sitting. The cross-sectional design does not establish temporal order, causality or an exercise prescription.

## 1 Introduction

### 1.1 Background

Depression is one of the leading contributors to the global burden of disease, affecting more than 300 million people worldwide, and is now the largest single contributor to disability (Huang et al., 2019). In China, the China Mental Health Survey estimated a lifetime prevalence of any depressive disorder of 6.8% and a 12-month prevalence of 3.6%, representing tens of millions of affected adults; fewer than one in ten of those with depressive disorders receives adequate treatment (Lu et al., 2021). Beyond diagnosed disorders, a substantial proportion of adults experience subthreshold depressive symptoms that are nonetheless associated with functional impairment, reduced quality of life, and an elevated risk of subsequent major depression. Identifying modifiable lifestyle factors that can prevent or alleviate depressive symptoms is therefore a public health priority.

Physical activity is among the most robustly supported behavioural determinants of mental health, with reviews in children and adolescents supporting a favourable role in mental health (Biddle and Asare, 2011) and reviews across depressive and anxiety disorders supporting this role in adults (Ströhle, 2009). Meta-analyses of prospective cohort studies report that physically active individuals have a 15–20% lower risk of developing depression (Mammen and Faulkner, 2013; Schuch et al., 2018; Pearce et al., 2022; Rebar et al., 2015), and large prospective cohort evidence, including the HUNT study, is consistent with this association (Harvey et al., 2018); reviews of adult observational evidence have similarly reported inverse associations between activity and likelihood of depression (Teychenne et al., 2008). Randomized controlled trials demonstrate that exercise reduces depressive symptoms to a degree comparable with established treatments (Kvam et al., 2016; Gordon et al., 2018; Cooney et al., 2013; Blumenthal et al., 2007; Schuch et al., 2016). Accordingly, current guidelines recommend that adults accumulate at least 150 minutes of moderate-intensity or 75 minutes of vigorous-intensity activity per week (Bull et al., 2020; Piercy et al., 2018), and even low volumes of activity have been associated with lower mortality and longer life expectancy (Wen et al., 2011). The protective mechanisms span neurobiological and psychosocial domains: exercise promotes neurotrophic factor secretion, modulates monoaminergic neurotransmission, and dampens stress reactivity (Dishman et al., 2006; Mikkelsen et al., 2017), while also conferring social contact, mastery, and self-efficacy (Firth et al., 2020). Physical activity has also been linked to cognitive health in longitudinal studies (Blondell et al., 2014). In China, where physical inactivity is highly prevalent (Guthold et al., 2018) and its economic burden is substantial (Ding et al., 2016), nationwide fitness has become a national strategy, providing a clear policy context for this research.

### 1.2 Research gap

A key question is whether the association between physical activity and mental health is linear or changes across the activity distribution. Chekroud et al. (2018) reported a nonlinear association in a large cross-sectional US sample, and Hamer et al. (2009) examined dose–response patterns in the Scottish Health Survey. These studies provide a rationale for examining flexible functional forms. However, findings based on exercise frequency, session duration or leisure-time activity cannot be converted directly into an optimum for total MET-min per week. Evidence from different study designs and activity measures should therefore be compared cautiously.

Evidence from a large Chinese survey can help examine whether the physical activity–depressive symptom association varies across activity levels. Interpretation requires attention to the sampling design, activity measurement, symptom scoring and activity domains. National geographical coverage does not by itself ensure that the complete-case analytic sample represents all Chinese adults, particularly when selection, nonresponse and weighting limitations remain.

### 1.3 Research question

We used the 2024 Chinese National Sports and Society Survey (CNSSS) to examine associations between total physical activity and depressive symptoms in community-dwelling adults. The continuous five-category symptom score was the primary outcome, and the recoded binary symptom indicator was an exploratory secondary outcome. Flexible modeling assessed the dose–response shape, with sensitivity analyses examining its dependence on model specification and the upper tail of the exposure distribution.

## 2 Materials and methods

### 2.1 Data source and sampling

Data were drawn from the Chinese National Sports and Society Survey (CNSSS), led by Wuhan Sports University and implemented by the survey research center at Renmin University of China. The supplied survey bulletin describes multistage probability sampling covering 31 provincial-level regions of mainland China, telephone interviewing and computer-assisted personal interviewing. The 2024 wave obtained 11,385 valid adult responses and contained 174 variables. The supplied dataset includes a weight variable but no identifiable PSU, stratum or replicate-weight fields; the detailed weight-construction protocol was not supplied. The data were obtained through the publicly accessible CNSSS download route, with accompanying data-use information. Ethics approval for the present secondary analysis was granted by the Human Experiment Ethics Committee of Nanjing Sport University (Reference No. RT-2026-30; 7 August 2026). This approval applies to the present analysis and is not presented as approval for the original 2024 CNSSS data collection. The available CNSSS materials do not specify the original ethics approval or exemption; these original-survey arrangements could not be verified from the available documentation. The survey population was residents aged 18 years or older living in mainland China in 2024, excluding Hong Kong, Macao and Taiwan residents, according to the supplied bulletin. This was a cross-sectional secondary analysis of the completed 2024 survey, distributed as the 2024–2025 release. Exact interview dates, stage-specific selection procedures and original invitation and nonresponse totals were not specified in the supplied release materials.

The primary analysis required valid activity data, all ten symptom items, specified covariates and a positive finite weight. Sequential exclusions were 7 for missing or invalid activity, 2 additional cases for missing symptom items and 713 additional cases for missing or invalid covariates; no further cases were excluded for weight validity. The final sample was 10,663, with 722 excluded (6.3%). Chronic-disease refusal was treated as missing rather than absence of disease, removing five cases retained in the original 10,668-person analysis. Included and excluded respondents were compared on available age, sex, education and household registration (Supplementary Analysis S1). An inverse-probability-of-complete-case sensitivity analysis was also performed. Study size was determined by the available released records and these analysis eligibility criteria; no prospective sample-size calculation was used for this secondary analysis.

### 2.2 Measures

#### 2.2.1 Depressive symptoms

The source questionnaire used five categories for experiences during the previous week (Radloff, 1977): every day, most of the time, sometimes or half of the time, not often, and rarely or none of the time (codes 1–5). For eight negative items, the symptom-oriented score was 6 minus the original code; for hopefulness and happiness, the original code was retained. Requiring all ten items produced the primary 10–50 score, with higher values indicating more symptoms. Cronbach's α was 0.865 among 11,383 item-complete respondents. This internal consistency does not validate the five-category structure against a diagnostic interview. For an exploratory screening outcome, each symptom-oriented item value 1, 2, 3, 4 or 5 was mapped to 0, 1, 2, 3 or 3, respectively; the resulting 0–30 total was dichotomized at ≥10. Equivalently, negative-item raw codes were mapped to 3, 3, 2, 1, 0 and positive-item codes to 0, 1, 2, 3, 3. Both mappings apply the same symptom-oriented severity collapse, but this study-specific transformation is not a validated conventional CES-D-10 protocol. Sensitivities used proportional scaling, 0.75 × (primary score −10), and an alternative complementary raw-code mapping for positive items (0, 0, 1, 2, 3). None of the resulting thresholds was interpreted as a clinical diagnosis or assumed to retain conventional screening properties (Andresen et al., 1994).

#### 2.2.2 Physical activity

Items A1b, A2b and A3b explicitly asked for cumulative activity time over the preceding week. Thus, for positive reported days, domain-specific weekly min were 60 × h + min, without an additional multiplication by days. Zero reported days were assigned zero min. Days outside 0–7, negative or special missing values, h outside 0–168, min outside 0–60 and domain durations above 10,080 min/week were invalid. A reported min value of 60 was retained as an equivalent additional h. Total MET-min/week was 8.0 × vigorous weekly min + 4.0 × moderate weekly min + 3.3 × weekly walking min, using the standard coefficients (Craig et al., 2003). Study volume categories were inactive (< 1), low (1– < 600), moderate (600– < 3,000) and high (≥3,000 MET-min/week). These categories do not reproduce the full IPAQ classification algorithm, directly measure guideline adherence or identify activity intensity. The survey did not partition these min into occupational, transport, domestic and leisure-time domains.

#### 2.2.3 Covariates

The primary model adjusted for age, sex, education, urban–rural household registration, marital status, annual personal income and chronic disease. Age was derived as 2024 minus birth year and modeled with restricted cubic splines at 21, 38, 54 and 76 years. Education comprised junior high or below, high school, associate college, bachelor and postgraduate categories. Agricultural and formerly agricultural registrations were classified as rural; non-agricultural and formerly non-agricultural registrations as urban; other or unknown registrations were missing. Household registration is not current residence. Income was transformed as ln(1 + income) after capping at the unweighted 99th percentile (500,000 yuan); negative or special missing responses were excluded. Chronic disease reflected any listed condition, with explicit refusal treated as missing. Sensitivity models excluded the emotional/mental-condition item from this composite. BMI was calculated as weight in jin × 0.5 divided by height in meters squared. Sleep duration was h + min/60; sitting duration was weekly h + min/60, divided by seven. Extended models included linear and quadratic terms for BMI, sleep duration and sitting duration, using BMI 10–60 kg/m^2^, sleep 0–16 h/night and sitting 0–24 h/day as admissible ranges. Smoking, alcohol use, employment/occupation and a direct general social-support scale were not available in the supplied questionnaire and 174-variable dataset. Sleep duration does not measure sleep quality, and the depressive-symptom sleep item was not reused as a covariate. These covariates were selected to account for demographic, socioeconomic and health differences that may relate to both activity and symptoms. Their inclusion does not establish a causal adjustment set, and additional health-behavior adjustment was assessed separately because these variables may also be mediators.

### 2.3 Statistical analysis

The supplied weight was treated as a relative sampling weight and normalized to mean one for numerical stability. Gaussian, modified Poisson and logistic coefficients solved weighted estimating equations. Variance was recalculated using the observation-level sandwich A^−^^1^[Σ u_i_u_i_^T^]A^−^^1^ × n/(n–p), where u_*i*_ = w_*i*_x_i_(y_i_–μ_i_), and A = Σ w_i_v_i_x_i_x_i_^T^; v_i_ was 1 for the identity-link Gaussian model, μ_*i*_ for the Poisson log-link model and μ_*i*_(1–μ_*i*_) for logistic regression. Squared weights enter the score outer products; each row remains one observation. This independent-observation approximation cannot recover unavailable clustering, stratification, finite-population or weight-estimation uncertainty. The Gaussian covariance was checked against WLS HC1, and coefficients and variances were verified to be invariant to rescaling the weights. Continuous symptom score was primary; the study-specific binary screen was exploratory and analyzed using modified Poisson prevalence ratios (PRs), with logistic odds ratios as sensitivity results. Categorical means and logistic screening probabilities were standardized over the same weighted empirical covariate distribution, with coefficient-based delta-method 95% intervals. Three score contrasts between active categories used two-sided Wald tests with Holm-adjusted *P* values. The dose–response model retained all analytic participants: inactivity was represented by an activity indicator, while active participants had a restricted cubic spline of log(MET), centered at 600 MET-min/week. Four knots were fixed before fitting at the weighted 5th, 35th, 65th and 95th percentiles among active participants: 297.00, 1,782.00, 4,860.00 and 30,465.88 MET-min/week. The two nonlinear basis coefficients were tested jointly against zero using a robust χ^2^ Wald test. Inactive and active predictions came from this common model and were standardized to the same population. The displayed active curve spans the observed positive minimum to the weighted 99th percentile; no numerical optimum or nadir estimate was reported. Sensitivities examined three/five knots, exclusion above unweighted 95th/99th activity percentiles, capping at the 99th percentile, excluding summed activity duration above 168 h/week, age and income form, sequential adjustment, extra BMI/sleep/sitting adjustment and alternative symptom recoding. A complete-case propensity model used available age, sex, education, log(1 + MET) and symptom score among eligible respondents; sampling weights were divided by predicted inclusion probabilities, bounded at 0.05–0.995. This selection-weight sensitivity assumes missingness is explainable by those measured predictors and does not establish missing at random. Analyses used Python (Python Software Foundation, Wilmington, Delaware, USA; https://www.python.org) with NumPy 2.5.3, pandas 3.0.6 and statsmodels 0.15.0. The spline specification was selected for this revision, not preregistered. Unadjusted categorical models included only an intercept and activity-group indicators and used the same analytic sample, supplied weights and sandwich variance as the adjusted models. Subgroup-specific models and effect-modification interactions were not performed. The activity boundaries were chosen as prespecified study-volume bands to describe the distribution and aid comparison, not as validated intensity or clinical thresholds.

## 3 Results

### 3.1 Sample characteristics

Among 10,663 analytic participants, unweighted counts were 244 inactive, 1,236 low, 4,921 moderate and 4,262 high. Weighted category shares were 5.1%, 10.5%, 38.9% and 45.5%, respectively. The weighted mean age was 46.8 years; 49.1% were female and 35.7% had urban household registration. The weighted primary symptom-score mean was 19.6 (SD 6.9); 40.8% met the study-specific exploratory screen, not a diagnostic definition. The unweighted MET median was 2,295 (IQR 1,074–4,697.9); the 90th, 95th and 99th percentiles were 10,232.8, 16,439.1 and 35,921.8, and the maximum was 55,998. The weighted median was 2,632.7 (IQR 1,059–6,930). Weights ranged from 0.0535 to 144.2839; the Kish weight-only effective sample size was approximately 615, which describes weight concentration rather than the complete survey-design effect. Table 1 reports all descriptive estimates for this single analytic sample. Variable-specific missing or invalid counts in the released sample, including the additional sensitivity-model covariates, are reported in Supplementary Table S4. Unadjusted categorical associations are reported in Supplementary Table S5; additional activity-group descriptive and outcome summaries appear in Supplementary Table S6. All these counts are unweighted, whereas summary measures and percentages are weighted.

**Table 1:** Weighted characteristics of the complete-case analytic sample overall and by activity volume.

Characteristic	Inactive	Low	Moderate	High	Total
Unweighted *n*	244	1,236	4,921	4,262	10,663
Age, mean (SD), years	43.8 (13.9)	46.1 (16.6)	46.5 (17.1)	47.6 (16.8)	46.8 (16.8)
Female, %	48.0	54.0	54.5	43.5	49.1
Urban household registration, %	26.1	40.6	38.9	32.9	35.7
Married, %	72.5	62.9	71.7	73.4	71.6
Any chronic disease, %	39.5	41.0	37.4	41.9	39.9
Education, %					
Junior high or below	82.9	62.9	57.0	67.2	63.6
High school	9.7	14.9	18.7	16.1	16.6
College (associate)	4.5	11.6	11.6	9.6	10.3
Bachelor	2.7	9.2	11.5	6.3	8.5
Postgraduate	0.2	1.3	1.2	0.8	1.0

All unweighted counts refer to the same 10,663 participants. Means, standard deviations and percentages are weighted. Urban refers to household registration rather than current residence. SD, standard deviation.

### 3.2 Physical activity and depressive symptoms

Relative to inactivity, adjusted continuous symptom-score differences were −3.11 (95% CI −5.94 to −0.27; *P* = 0.032) for low, −3.41 (−6.13 to −0.69; *P* = 0.014) for moderate and −2.20 (−4.96 to 0.57; *P* = 0.120) for high activity. Population-standardized means were 22.23 (19.58–24.89), 19.13 (18.17–20.08), 18.83 (18.27–19.38) and 20.04 (19.22–20.85), respectively (Figure 1a; Table 2). Moderate minus low activity was −0.30 (95% CI −1.38 to 0.78; Holm-adjusted *P* = 0.587), providing no clear evidence that moderate activity was superior. High minus moderate was 1.21 (0.23–2.19; Holm-adjusted *P* = 0.045), whereas high minus low was 0.91 (−0.38 to 2.20; Holm-adjusted *P* = 0.331). These are adjusted associations, not treatment effects.

**Figure 1:**
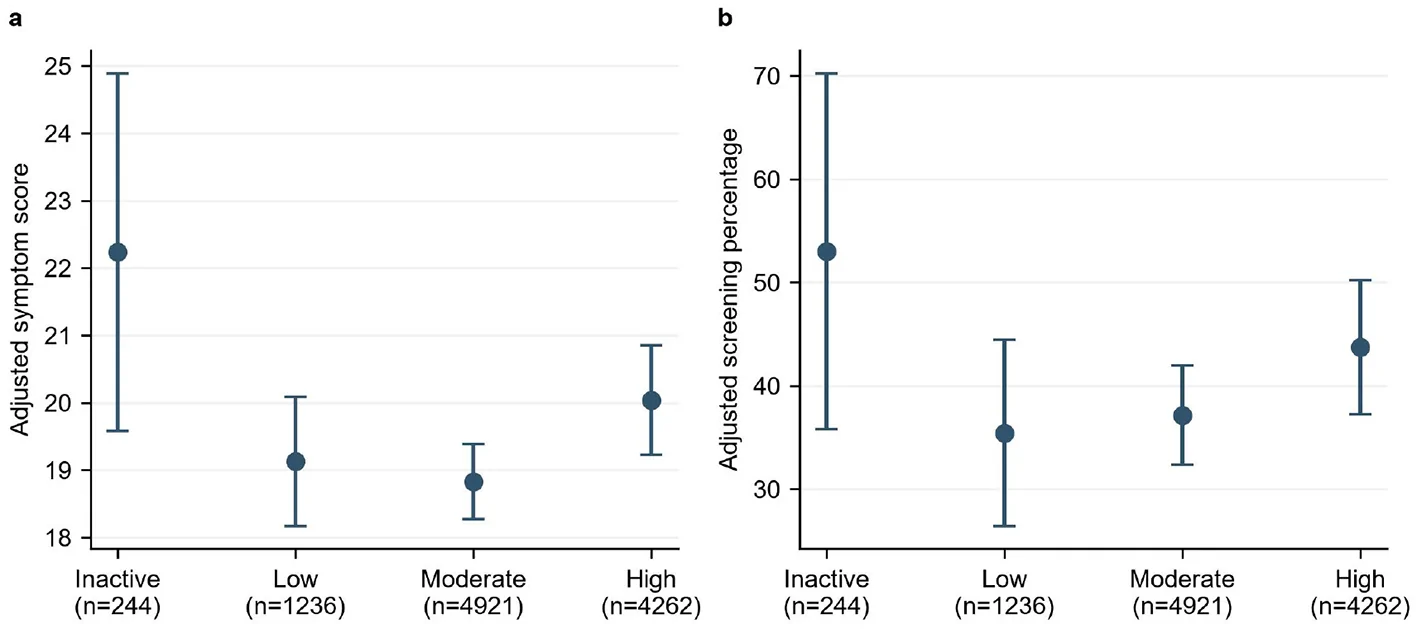
Population-standardized estimates by activity volume (*n* = 10,663). **(a)** Primary five-category symptom score (10–50) from the weighted Gaussian model. **(b)** Study-specific exploratory screening percentage from the logistic sensitivity model. Points show means or probabilities; bars are coefficient-based delta-method 95% intervals using the observation-level sandwich covariance and fixed empirical covariate distribution. Each group is standardized to the same weighted population; unweighted group counts are shown. Adjustment is as described in Section 2.3.

**Table 2:** Adjusted categorical associations with the primary score and exploratory screening outcome.

Activity volume	Adjusted score mean (95% CI)	Adjusted screen % (95% CI)	Score difference β (95% CI)	Adjusted PR (95% CI)
Inactive (< 1)	22.23 (19.58 to 24.89)	53.0 (35.8 to 70.2)	Reference	Reference
Low (1– < 600)	19.13 (18.17 to 20.08)	35.4 (26.4 to 44.5)	−3.11 (−5.94 to −0.27)	0.68 (0.45 to 1.03)
Moderate (600– < 3,000)	18.83 (18.27 to 19.38)	37.2 (32.3 to 42.0)	−3.41 (−6.13 to −0.69)	0.71 (0.50 to 1.01)
High (≥3,000)	20.04 (19.22 to 20.85)	43.8 (37.3 to 50.2)	−2.20 (−4.96 to 0.57)	0.84 (0.59 to 1.19)

All models use the same complete-case sample and adjustment set in Section 2.3. Score means are Gaussian-standardized; screening percentages are logistic-standardized. β denotes score difference from inactivity; PR denotes modified Poisson prevalence ratio. Parentheses give 95% intervals using the observation-level sandwich approximation. Continuous and binary outcomes use distinct scoring metrics. Inactivity is the reference. CI, confidence interval; MET, metabolic equivalent.

For the exploratory binary screen, adjusted prevalence ratios were 0.68 (95% CI 0.45–1.03; *P* = 0.069), 0.71 (0.50–1.01; *P* = 0.059) and 0.84 (0.59–1.19; *P* = 0.323) for low, moderate and high activity, respectively. All intervals included 1 (Figure 2; Table 2). Logistic sensitivity ORs were 0.45 (0.19–1.09), 0.49 (0.22–1.09) and 0.66 (0.30–1.48). Logistic-standardized screening percentages were 53.0% (35.8–70.2), 35.4% (26.4–44.5), 37.2% (32.3–42.0) and 43.8% (37.3–50.2) (Figure 1b). The binary estimates were imprecise and did not establish identical ordering or a single best activity category.

**Figure 2:**
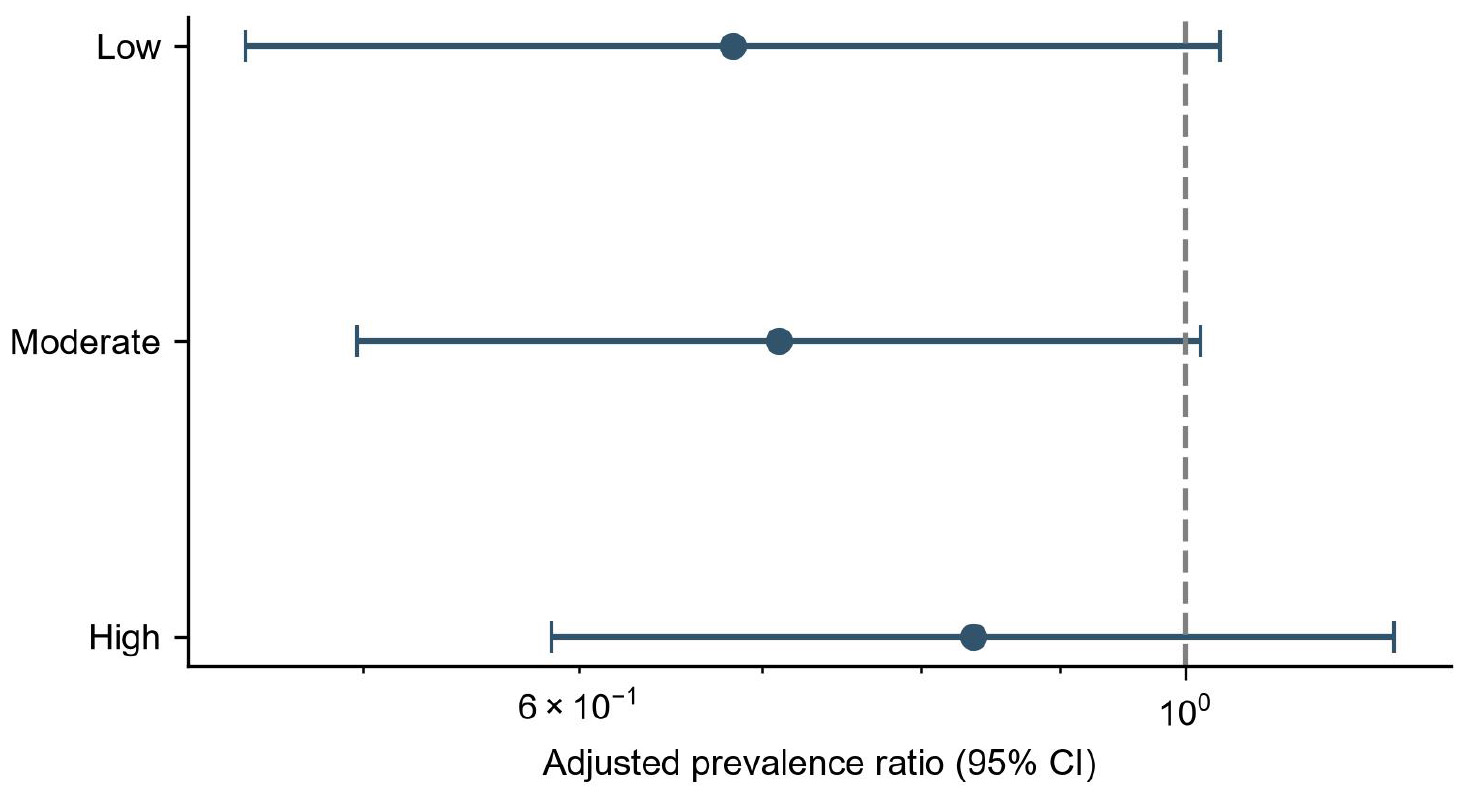
Adjusted prevalence ratios for the exploratory recoded symptom screen, estimated by weighted modified Poisson equations with observation-level robust sandwich 95% intervals. Inactivity is the reference; the dashed line denotes PR = 1. The analytic sample comprised 10,663 participants. These ratios are not odds ratios or causal risk reductions.

### 3.3 Nonlinear dose–response shape

The two-part restricted cubic spline model gave a nonlinear-term Wald χ^2^ of 6.75 on 2 degrees of freedom (P = 0.034). The active curve declined across lower volumes and rose across part of the upper distribution (Figure 3). The standardized inactive estimate from the same model was 22.25 (95% CI 19.59–24.90). Evidence for curvature depended on specification: *P* was 0.309 with three knots and 0.045 with five knots; exclusion above the 95th percentile gave *P* = 0.063, compared with *P* = 0.023 after exclusion above the 99th percentile. Additional BMI, sleep-duration and sitting-duration adjustment (*n* = 10,623) gave *P* = 0.019. Binary screening percentages changed to 26.0% with proportional scaling and 33.2% with complementary raw-code recoding. The propensity-weighted complete-case sensitivity gave nonlinear *P* = 0.032. Detailed sensitivity results and included–excluded comparisons appear in the Supplementary Analyses. A stable numerical minimum, optimal dose, harmful high-activity effect or causal mechanism was not inferred.

**Figure 3:**
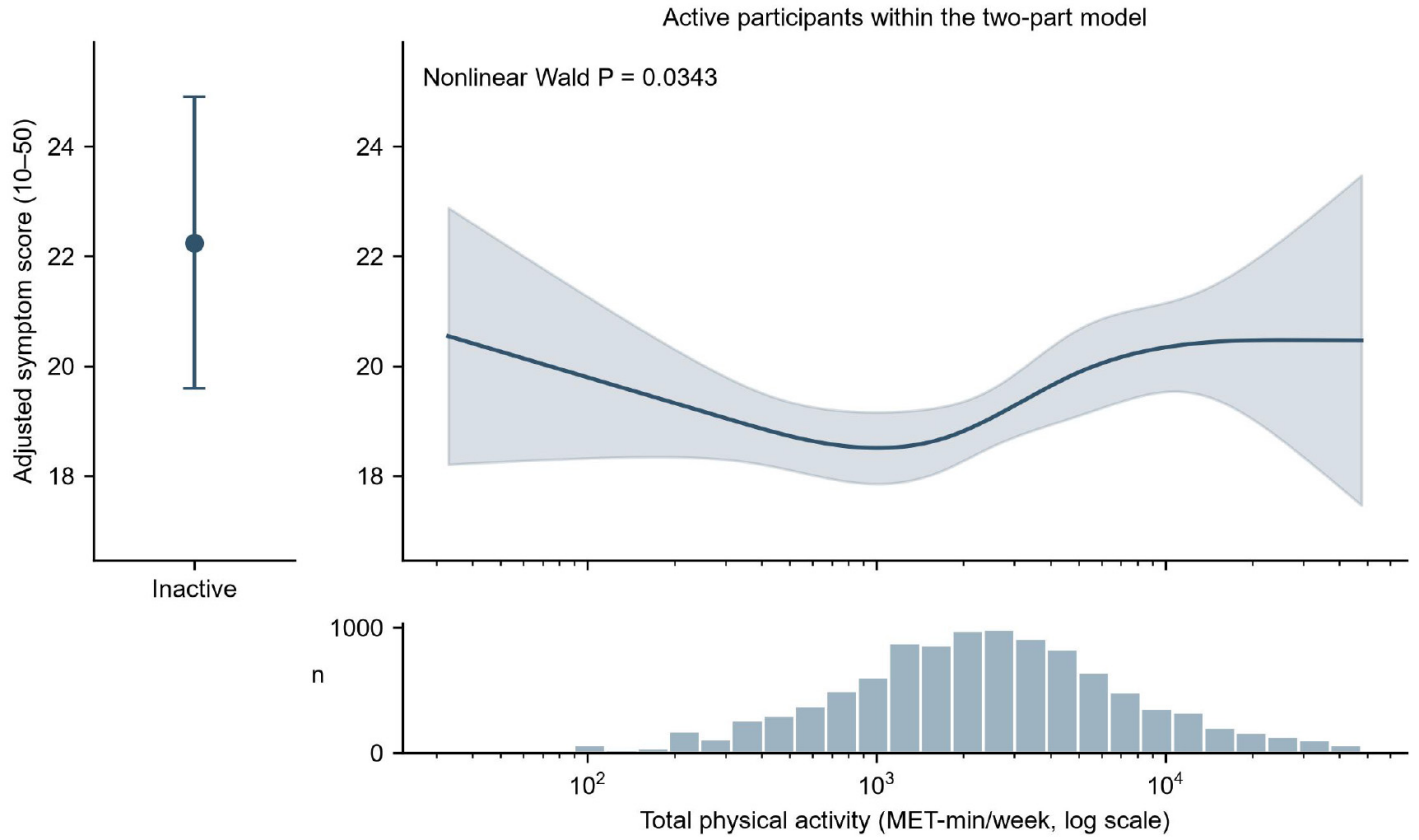
Common two-part model for inactivity and activity, with population-standardized primary symptom scores and observation-level sandwich 95% intervals. The left point represents inactivity; the right curve represents positive activity on a log scale. They use the same adjustment model and weighted standardization population. The histogram shows unweighted active observations within the plotted range, from the observed positive minimum to the weighted 99th percentile. Knots and the joint two-term robust Wald test are specified in Section 2.3. No optimal dose is highlighted. The inactive category and active curve are distinct components rather than one continuous curve through zero.

## 4 Discussion

### 4.1 Principal findings

Low and moderate activity were associated with lower continuous symptom scores than inactivity, with no clear difference between low and moderate activity. The high-group point estimate was also lower than inactivity, but its interval included no difference; it cannot establish a benefit or harm. The exploratory binary estimates were imprecise, and all primary PR intervals included 1. The main spline showed evidence of possible curvature, but this weakened under alternative knots and upper-tail exclusion. Consequently, neither a stable J-shaped relationship nor a precise optimal dose is established. Reverse causation and self-selection remain plausible.

### 4.2 Comparison with previous studies

Prospective and intervention findings provide context for physical activity and depressive symptoms (Mammen and Faulkner, 2013; Schuch et al., 2018; Pearce et al., 2022). Cross-sectional reports by Chekroud et al. (2018) and Hamer et al. (2009) motivate flexible modeling, but differences in activity domains, outcome measures and designs prevent direct conversion of their frequency or duration findings into an optimum for total MET-min/week. The present reanalysis adds an exploratory assessment in Chinese adults while showing that nonlinear evidence and screening estimates depend on measurement and modeling choices. It does not establish a common optimum across populations or uniformly superior moderate activity.

### 4.3 Possible mechanisms

If curvature reflects a reproducible association, neurobiological and psychosocial pathways discussed in previous work may be relevant (Dishman et al., 2006; Firth et al., 2020), but none was directly tested here. Total activity does not distinguish occupational, transport, domestic and leisure-time activity, so high volume cannot diagnose excessive training or identify high-intensity exercise (Kreher and Schwartz, 2012). Occupational exertion, socioeconomic disadvantage and work-related stress are plausible alternatives to an overtraining explanation (Ekkekakis et al., 2011; Reed and Buck, 2009); domain differences discussed by Holtermann et al. (2018) offer context rather than direct evidence for a depression mechanism. Reverse causation, self-selection, self-report error and influential upper-tail observations may also shape the curve. These remain hypotheses, not mechanisms demonstrated by the survey.

### 4.4 Limitations

First, the cross-sectional design cannot establish temporal order or causality and does not resolve reverse causation or self-selection. Second, self-reported activity and symptoms are vulnerable to recall and social-desirability bias; IPAQ-based activity reporting may overestimate volume and distort the upper-tail shape. Third, the five-category symptom format and its study-specific recoding are not validated against diagnostic interviews, and neither internal consistency nor an established cut-off from another scoring format resolves this issue. Alternative recoding changed screening percentages materially. Fourth, occupational, transport, domestic and leisure-time min could not be separated. Smoking, alcohol, employment/occupation and direct general social support were absent from the supplied questionnaire/data; sleep duration did not measure sleep quality. Additional BMI, sleep and sitting adjustment may also involve mediating variables and cannot eliminate confounding. Fifth, complete-case exclusion may select a different population; propensity weighting addresses only measured selection predictors under an unverified missingness assumption. Sixth, strata, PSU and replicate weights were unavailable, so observation-level robust variance does not recover the full sampling design; the construction and calibration of the supplied weights could not be independently verified from the available documentation. Highly concentrated weights yielded a weight-only effective sample size around 615. Finally, curvature was sensitive to knot specification and upper-tail exclusion; a precise minimum and national generalizability should not be assumed. Household registration is not current residence, and the results do not generalize directly to athletes or coaches. Self-report overestimation may inflate the upper exposure tail, and residual confounding and selection may alter association direction or magnitude; their net effect cannot be quantified from these data. The exact original sampling stages, fieldwork dates and original nonresponse rate could not be verified.

### 4.5 Implications

These results are exploratory evidence concerning physical activity and symptom burden in community-dwelling adults. They do not support a new activity prescription, an optimal numerical dose or an activity ceiling inferred from a cross-sectional upper-tail upturn. Existing guidelines remain the basis for public-health advice. Longitudinal and intervention studies should address temporality, activity domains and measurement validity and assess whether any nonlinear pattern is reproducible.

## 5 Conclusion

Low and moderate activity were associated with lower continuous depressive-symptom scores than inactivity, without a clear difference between those two categories. The possible nonlinear pattern depended on the spline and upper-tail specification, and exploratory binary estimates were imprecise. A stable J-shaped relationship or optimal dose was not established. The high-group estimates do not demonstrate greater harm than inactivity; high activity volume is distinct from intensity, and an inactivity category is not equivalent to prolonged sitting. No causal effect or new exercise prescription can be inferred.

## Data Availability

The raw data supporting the conclusions of this article will be made available by the authors, without undue reservation.

## Supplementary material

Supplementary Analysis S1 Comparison of included and excluded respondents on age, sex, education, and household registration.

Supplementary Table S4 Variable-specific missing or invalid counts in the released sample, including the additional sensitivity-model covariates.

Supplementary Table S5 Unadjusted categorical associations between activity volume and depressive symptoms.

Supplementary Table S6 Additional activity-group descriptive and outcome summaries.
