# Supplementary material for: Physical activity and depressive symptoms among Chinese adults a cross sectional analysis
Source: Front Psychol. 2026 Oct 27;17:1989343. doi: 10.3389/fpsyg.2026.1989343 (PMC13747850; doi:10.3389/fpsyg.2026.1989343)
Supplement: Supplementary file 1 [file Table_1.docx]

Supplementary Analyses

Sensitivity analyses assessed the dependence of the estimates on analytical specification. The observational variance approximation, unverified weight-construction details and lack of diagnostic validation remain limitations. Alternative models assess specification sensitivity, not independent replication.

Supplementary Analysis S1 Included and excluded respondents

Age: included 46.8 years (available n = 10663); excluded 45.6 years (available n = 572).

Female: included 49.1 % (available n = 10663); excluded 52.2 % (available n = 722).

Urban hukou: included 35.7 % (available n = 10663); excluded 27.7 % (available n = 523).

College or above: included 19.8 % (available n = 10663); excluded 14.6 % (available n = 697).

Values are sampling-weighted and use available observations for each characteristic; excluded denominators therefore vary. Education refers to associate college or higher. These differences do not establish the missingness mechanism.

Supplementary Analysis S2 Model and exposure sensitivities

Demographic adjustment (n = 10663): adjusted score differences versus inactivity, low -2.98 (-5.88 to -0.09); moderate -3.70 (-6.53 to -0.88); high -2.33 (-5.19 to 0.53); nonlinear Wald P = 0.003.

Socioeconomic adjustment (n = 10663): adjusted score differences versus inactivity, low -3.11 (-6.01 to -0.22); moderate -3.51 (-6.29 to -0.72); high -2.21 (-5.03 to 0.61); nonlinear Wald P = 0.029.

Primary model (n = 10663): adjusted score differences versus inactivity, low -3.11 (-5.94 to -0.27); moderate -3.41 (-6.13 to -0.69); high -2.20 (-4.96 to 0.57); nonlinear Wald P = 0.034.

Primary model on expanded-model sample (n = 10623): adjusted score differences versus inactivity, low -3.12 (-5.97 to -0.28); moderate -3.43 (-6.16 to -0.70); high -2.20 (-4.98 to 0.57); nonlinear Wald P = 0.034.

Additional BMI sleep and sitting adjustment (n = 10623): adjusted score differences versus inactivity, low -2.86 (-5.51 to -0.21); moderate -3.22 (-5.79 to -0.66); high -1.89 (-4.48 to 0.70); nonlinear Wald P = 0.019.

Linear age (n = 10663): adjusted score differences versus inactivity, low -3.08 (-5.90 to -0.27); moderate -3.37 (-6.08 to -0.66); high -2.16 (-4.92 to 0.60); nonlinear Wald P = 0.030.

Income not winsorized (n = 10663): adjusted score differences versus inactivity, low -3.11 (-5.94 to -0.27); moderate -3.41 (-6.13 to -0.69); high -2.20 (-4.96 to 0.57); nonlinear Wald P = 0.034.

Chronic disease excludes mental or emotional condition (n = 10663): adjusted score differences versus inactivity, low -3.16 (-6.03 to -0.29); moderate -3.47 (-6.23 to -0.72); high -2.22 (-5.02 to 0.58); nonlinear Wald P = 0.031.

Exclude above unweighted MET 99th percentile (n = 10556): adjusted score differences versus inactivity, low -3.12 (-5.97 to -0.28); moderate -3.41 (-6.13 to -0.68); high -2.05 (-4.81 to 0.72); nonlinear Wald P = 0.023.

Exclude above unweighted MET 95th percentile (n = 10129): adjusted score differences versus inactivity, low -2.96 (-5.83 to -0.08); moderate -3.26 (-6.06 to -0.47); high -2.49 (-5.30 to 0.32); nonlinear Wald P = 0.063.

Cap MET at unweighted 99th percentile (n = 10663): adjusted score differences versus inactivity, low -3.11 (-5.94 to -0.27); moderate -3.41 (-6.13 to -0.69); high -2.20 (-4.96 to 0.57); nonlinear Wald P = 0.030.

Exclude activity duration over 168 hours weekly (n = 10648): adjusted score differences versus inactivity, low -3.11 (-5.95 to -0.27); moderate -3.38 (-6.11 to -0.66); high -2.20 (-4.97 to 0.56); nonlinear Wald P = 0.035.

Alternative 3 knots (n = 10663): adjusted score differences versus inactivity, low -3.11 (-5.94 to -0.27); moderate -3.41 (-6.13 to -0.69); high -2.20 (-4.96 to 0.57); nonlinear Wald P = 0.309.

Alternative 5 knots (n = 10663): adjusted score differences versus inactivity, low -3.11 (-5.94 to -0.27); moderate -3.41 (-6.13 to -0.69); high -2.20 (-4.96 to 0.57); nonlinear Wald P = 0.045.

Collapsed five-category 0 to 30 score (n = 10663): adjusted score differences versus inactivity, low -2.15 (-4.55 to 0.25); moderate -2.44 (-4.75 to -0.14); high -1.42 (-3.76 to 0.91); nonlinear Wald P = 0.041.

Complementary raw-code recoding 0 to 30 (n = 10663): adjusted score differences versus inactivity, low -2.33 (-4.69 to 0.03); moderate -2.65 (-4.91 to -0.40); high -1.59 (-3.88 to 0.69); nonlinear Wald P = 0.018.

Inverse-probability-of-complete-case sensitivity (n = 10663): adjusted score differences versus inactivity, low -2.87 (-5.72 to -0.02); moderate -3.17 (-5.91 to -0.43); high -1.97 (-4.75 to 0.81); nonlinear Wald P = 0.032.

Unless stated otherwise, knots remain fixed at the primary locations and the original sampling weights are retained. Capping at 35,921.82 and exclusions above 35,921.82 or 16,439.10 MET-min/week use unweighted analytic-sample percentiles. Alternative spline knots use weighted active log-MET percentiles 10, 50, 90 for three knots and 5, 27.5, 50, 72.5, 95 for five knots. Expanded models include quadratic BMI, sleep and sitting terms; a primary model on the same 10,623 cases separates sample change from adjustment change. Score-recoding models use different scales, so their coefficients should not be directly compared numerically with the 10–50-score coefficients.

Supplementary Analysis S3 Exploratory screening recoding

Common symptom-category collapse: weighted screening percentage 40.8%; adjusted PRs versus inactivity, low 0.68 (0.45 to 1.03); moderate 0.71 (0.50 to 1.01); high 0.84 (0.59 to 1.19).

Proportional scaling: weighted screening percentage 26.0%; adjusted PRs versus inactivity, low 0.60 (0.34 to 1.03); moderate 0.62 (0.39 to 0.98); high 0.80 (0.51 to 1.27).

Complementary positive-item raw-code mapping: weighted screening percentage 33.2%; adjusted PRs versus inactivity, low 0.63 (0.39 to 1.02); moderate 0.67 (0.44 to 1.02); high 0.84 (0.55 to 1.28).

All binary models use the 10,663-person analytic sample and threshold ≥10 on the relevant study-specific 0–30 scale. These assumptions materially change screening percentages and do not validate a diagnosis. Logistic OR sensitivity estimates are reported in Section 3.2.

## Supplementary Table S4 Variable availability

Counts below are unweighted and refer to missing or invalid derived analysis values after the rules in Section 2.2. Counts overlap across variables and must not be summed to reproduce sequential exclusions. The primary score and exploratory screen use the same ten items. Additional BMI, sleep and sitting requirements jointly leave 10,623 of the 10,663 primary analytic cases.

| **Analysis variable** | **Unavailable in release n = 11,385** | **Unavailable in primary sample n = 10,663** |
| --- | --- | --- |
| Total physical activity | 7 | 0 |
| Primary symptom score | 2 | 0 |
| Exploratory symptom screen | 2 | 0 |
| Age | 150 | 0 |
| Sex | 0 | 0 |
| Education | 25 | 0 |
| Household registration | 199 | 0 |
| Marital status | 21 | 0 |
| Annual personal income | 462 | 0 |
| Chronic disease | 7 | 0 |
| Sampling weight | 0 | 0 |
| BMI | 36 | 17 |
| Sleep duration | 21 | 19 |
| Sitting duration | 9 | 4 |

## Supplementary Table S5 Unadjusted categorical associations

All models use the same 10,663 cases as Table 2. They include only an intercept and activity category, retain the supplied sampling weights and use observation-level sandwich 95% confidence intervals. No demographic or health covariates are included. Inactivity is the reference. Adjusted estimates and the adjustment set are reported in Table 2 and Section 2.3; exploratory prevalence ratios are not causal risk reductions.

| **Activity group** | **Unadjusted score difference (95% CI)** | **Unadjusted screening PR (95% CI)** |
| --- | --- | --- |
| Low | -2.97 (-5.87 to -0.07) | 0.69 (0.47 to 1.03) |
| Moderate | -3.69 (-6.50 to -0.88) | 0.69 (0.49 to 0.96) |
| High | -2.48 (-5.36 to 0.40) | 0.80 (0.56 to 1.13) |

## Supplementary Table S6 Additional group summaries

The descriptive values below use the same primary sample. Means, standard deviations and percentages are sampling-weighted; counts are unweighted. Income is uncapped annual personal income in yuan, whereas the model uses the capped log-transformed value described in Section 2.2. The screen is study-specific and exploratory.

| **Group** | **Activity mean (SD), MET-min/week** | **Score mean (SD)** | **Income mean (SD), yuan** | **Screen count / group n; weighted %** |
| --- | --- | --- | --- | --- |
| Inactive | 0.0 (0.0) | 22.46 (8.71) | 35,249 (37,653) | 144 / 244; 54.1% |
| Low | 333.4 (165.5) | 19.49 (6.11) | 48,111 (122,155) | 664 / 1236; 37.5% |
| Moderate | 1666.7 (663.4) | 18.77 (6.40) | 48,786 (92,683) | 2522 / 4921; 37.2% |
| High | 13592.4 (11997.9) | 19.97 (7.05) | 45,541 (112,764) | 1991 / 4262; 43.1% |
